# 2-[4-(Tri­fluoro­meth­yl)phenyl­sulfan­yl]benzoic acid

**DOI:** 10.1107/S1600536813028778

**Published:** 2013-10-26

**Authors:** Thammarse S. Yamuna, Jerry P. Jasinski, Brian J. Anderson, H.S. Yathirajan, Manpreet Kaur

**Affiliations:** a Department of Studies in Chemistry, University of Mysore, Manasagangotri, Mysore 570 006, India; b Department of Chemistry, Keene State College, 229 Main Street, Keene, NH 03435-2001, USA

## Abstract

In the title compound, C_14_H_9_F_3_O_2_S, the dihedral angle between the mean planes of the benzene rings is 88.7 (2)°. The carb­oxy­lic acid group is twisted by 13.6 (7)° from the mean plane of its attached aromatic ring. One of the F atoms of the tri­fluoro­methyl group is disordered over two sites in a 0.61 (7):0.39 (7) ratio. In the crystal, inversion dimers linked by pairs of O—H⋯O hydrogen bonds generate *R*_2_^2^(8) loops. Weak C—H⋯F inter­actions are also observed.

## Related literature

For background to the neuroleptic agent flupentixol (systematic name: (*EZ*)-2-[4-[3-[2-(tri­fluoro­meth­yl)thio­xan­then-9-yl­idene]prop­yl]piperazin-1-yl]ethanol), see: Young *et al.* (1976 ▶). For related structures, see: Post *et al.* (1975*a* ▶,*b* ▶); Siddegowda *et al.* (2011*a* ▶,*b* ▶).
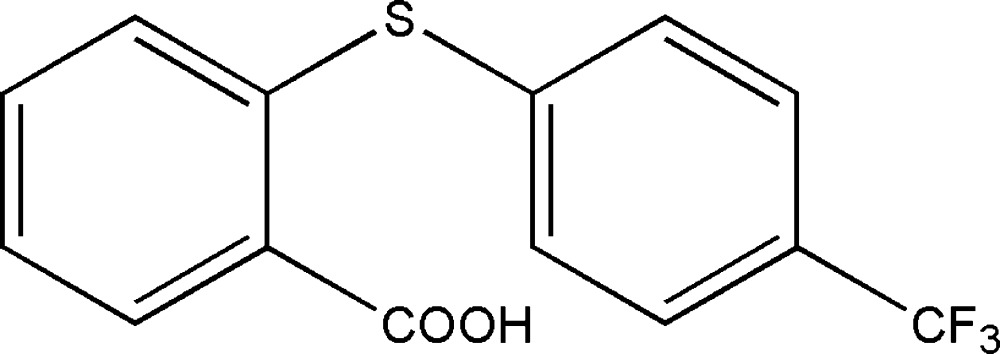


## Experimental

### 

#### Crystal data

C_14_H_9_F_3_O_2_S
*M* *_r_* = 298.28
Triclinic, 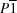

*a* = 7.3071 (5) Å
*b* = 8.0790 (7) Å
*c* = 11.3878 (11) Å
α = 82.678 (8)°
β = 83.642 (7)°
γ = 72.309 (7)°
*V* = 633.41 (10) Å^3^
*Z* = 2
Cu *K*α radiation
μ = 2.63 mm^−1^
*T* = 173 K
0.24 × 0.22 × 0.12 mm

#### Data collection

Agilent Gemini EOS diffractometer
Absorption correction: multi-scan (*CrysAlis PRO* and *CrysAlis RED*; Agilent, 2012 ▶) *T*_min_ = 0.848, *T*_max_ = 1.000
3701 measured reflections
2419 independent reflections
2049 reflections with *I* > 2σ(*I*)
*R*_int_ = 0.038

#### Refinement

*R*[*F* ^2^ > 2σ(*F* ^2^)] = 0.054
*wR*(*F* ^2^) = 0.155
*S* = 1.04
2419 reflections
193 parameters
H-atom parameters constrained
Δρ_max_ = 0.71 e Å^−3^
Δρ_min_ = −0.38 e Å^−3^

### 

Data collection: *CrysAlis PRO* (Agilent, 2012 ▶); cell refinement: *CrysAlis PRO* (Agilent, 2012 ▶); data reduction: *CrysAlis RED*; program(s) used to solve structure: *SUPERFLIP* (Palatinus & Chapuis, 2007 ▶); program(s) used to refine structure: *SHELXL2012* (Sheldrick, 2008 ▶); molecular graphics: *OLEX2* (Dolomanov *et al.*, 2009 ▶); software used to prepare material for publication: *OLEX2*.

## Supplementary Material

Crystal structure: contains datablock(s) I. DOI: 10.1107/S1600536813028778/hb7150sup1.cif

Structure factors: contains datablock(s) I. DOI: 10.1107/S1600536813028778/hb7150Isup2.hkl

Supplementary material file. DOI: 10.1107/S1600536813028778/hb7150Isup3.cml

Additional supplementary materials:  crystallographic information; 3D view; checkCIF report

## Figures and Tables

**Table 1:** Hydrogen-bond geometry (Å, °)

*D*—H⋯*A*	*D*—H	H⋯*A*	*D*⋯*A*	*D*—H⋯*A*
O2—H2⋯O1^i^	0.82	1.86	2.677 (3)	175
C6—H6⋯F3^ii^	0.93	2.59	3.319 (10)	136
C6—H6⋯F3*A* ^ii^	0.93	2.50	3.294 (16)	144

Symmetry codes: (i) 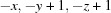; (ii) 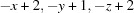.

**Fig. 1.**
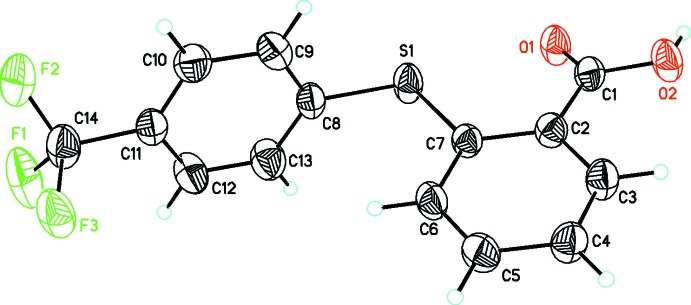
ORTEP drawing of (I) showing 50% probability displacement ellipsoids. Disordered atom, F3A (0.39 (7) occupancy) has been removed for clarity.

**Fig. 2.**
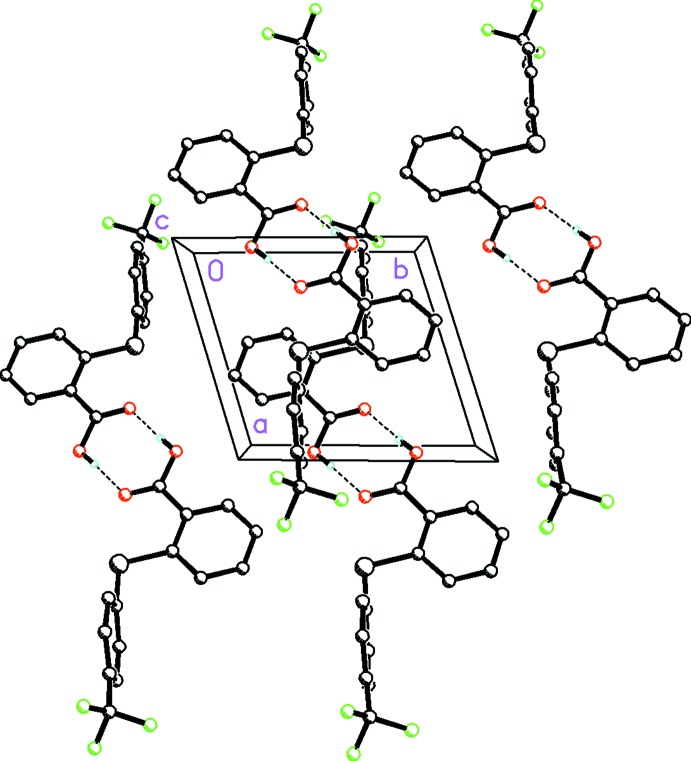
Molecular packing for (I) viewed along the c axis. Dashed lines indicate O—H···O intermolecular hydrogen bonds which link the molecules into dimers with R22[8] graph-set motifs and influence the crystal packing. Disordered atom, F3A (0.39 (7) occupancy) and H atoms not involved in hydrogen bonding have been removed for clarity.

## supplementary crystallographic information

### 1. Comment

The title compound, (I), is a starting material for the synthesis of flupentixol, a well-documented neuroleptic (Young *et al.*, 1976). The crystal structures of α-flupentixol (Post *et al.*, 1975*a*) and β-flupentixol (Post *et al.*, 1975*b*) have been reported. As part of our onging studies in this area (Siddegowda *et al.*, 2011*a*,*b*), we now describe the crystal structure of (I).

The dihedral angle between the mean planes of the phenyl rings is 88.7 (2)°. The carboxylic acid group (C2/C1/O2/O1) is twisted by 13.6 (7)° from the mean plane of the adjacent benzene ring (C2–C7). Disorder was modeled over two sets of sites for one fluorine atom (F3), of the trifluoromethyl group with an occupancy ratio of 0.61 (7) : 0.39 (7). In the crystal, O—H···O hydrogen bonds (Table 1) link the molecules into dimers with R_2_^2^[8] graph-set motifs (Fig. 2). Weak C—H···F interactions are also observed.

### 2. Experimental

The title compound was obtained as a gift sample from R. L. Fine chemicals, Bengaluru. It was dissolved in 15 ml of mixture of acetonitrile and dimethyl sulphoxide (1:2), stirred for 10 minutes at room temperature. After few days, irregular colourless crystals were formed by slow evaporation of the solvent mixture (m.p: 413-418 K).

### 3. Refinement

All of the H atoms were placed in their calculated positions and then refined using the riding model with Atom—H lengths of 0.93Å (CH) or 0.82Å (OH). Isotropic displacement parameters for these atoms were set to 1.2 (CH) or 1.5 (OH) times *U*_eq_ of the parent atom. Disorder was modeled over two sets of sites for one fluorine atom (F3) in the trifluoromethyl group with an occupancy ratio of 0.61 (7) : 0.39 (7). Idealised tetrahderal OH refined as rotating group.

### Crystal data

**Table:**

C_14_H_9_F_3_O_2_S	*Z* = 2
*M**_r_* = 298.28	*F*(000) = 304
Triclinic, *P*1	*D*_x_ = 1.564 Mg m^−^^3^
*a* = 7.3071 (5) Å	Cu *K*α radiation, λ = 1.54184 Å
*b* = 8.0790 (7) Å	Cell parameters from 1645 reflections
*c* = 11.3878 (11) Å	θ = 5.8–72.1°
α = 82.678 (8)°	µ = 2.63 mm^−^^1^
β = 83.642 (7)°	*T* = 173 K
γ = 72.309 (7)°	Irregular, colourless
*V* = 633.41 (10) Å^3^	0.24 × 0.22 × 0.12 mm

### Data collection

**Table:**

Agilent Gemini EOS diffractometer	2419 independent reflections
Radiation source: Enhance (Cu) X-ray Source	2049 reflections with *I* > 2σ(*I*)
Detector resolution: 16.0416 pixels mm^-1^	*R*_int_ = 0.038
ω scans	θ_max_ = 72.2°, θ_min_ = 5.8°
Absorption correction: multi-scan (*CrysAlis PRO* and *CrysAlis RED*; Agilent, 2012)	*h* = −8→6
*T*_min_ = 0.848, *T*_max_ = 1.000	*k* = −9→6
3701 measured reflections	*l* = −13→14

### Refinement

**Table:**

Refinement on *F*^2^	Hydrogen site location: inferred from neighbouring sites
Least-squares matrix: full	H-atom parameters constrained
*R*[*F*^2^ > 2σ(*F*^2^)] = 0.054	*w* = 1/[σ^2^(*F*_o_^2^) + (0.0889*P*)^2^ + 0.2312*P*] where *P* = (*F*_o_^2^ + 2*F*_c_^2^)/3
*wR*(*F*^2^) = 0.155	(Δ/σ)_max_ < 0.001
*S* = 1.04	Δρ_max_ = 0.71 e Å^−^^3^
2419 reflections	Δρ_min_ = −0.38 e Å^−^^3^
193 parameters	Extinction correction: *SHELXL2012* (Sheldrick, 2008), Fc^*^=kFc[1+0.001xFc^2^λ^3^/sin(2θ)]^-1/4^
0 restraints	Extinction coefficient: 0.0044 (14)
Primary atom site location: structure-invariant direct methods	

### Special details

**Table:**

Geometry. All esds (except the esd in the dihedral angle between two l.s. planes) are estimated using the full covariance matrix. The cell esds are taken into account individually in the estimation of esds in distances, angles and torsion angles; correlations between esds in cell parameters are only used when they are defined by crystal symmetry. An approximate (isotropic) treatment of cell esds is used for estimating esds involving l.s. planes.

### Fractional atomic coordinates and isotropic or equivalent isotropic displacement parameters (Å^2^)

**Table:**

	*x*	*y*	*z*	*U*_iso_*/*U*_eq_	Occ. (<1)
S1	0.51402 (10)	0.35821 (8)	0.70404 (6)	0.0398 (3)	
F1	1.2832 (3)	0.1041 (3)	1.0280 (2)	0.0764 (7)	
F2	1.0648 (3)	0.1392 (4)	1.16595 (19)	0.0849 (8)	
F3	1.1664 (14)	0.3549 (10)	1.0613 (12)	0.054 (2)	0.61 (7)
F3A	1.094 (10)	0.3613 (16)	1.106 (6)	0.100 (14)	0.39 (7)
O1	0.2047 (3)	0.4458 (2)	0.56512 (17)	0.0405 (5)	
O2	−0.0035 (3)	0.7122 (2)	0.54064 (19)	0.0427 (5)	
H2	−0.0646	0.6592	0.5121	0.064*	
C1	0.1620 (4)	0.6021 (3)	0.5733 (2)	0.0332 (5)	
C2	0.2907 (4)	0.6861 (3)	0.6177 (2)	0.0316 (5)	
C3	0.2487 (4)	0.8678 (3)	0.5973 (2)	0.0376 (6)	
H3	0.1387	0.9315	0.5595	0.045*	
C4	0.3668 (4)	0.9550 (3)	0.6319 (2)	0.0396 (6)	
H4	0.3375	1.0757	0.6172	0.048*	
C5	0.5291 (4)	0.8595 (4)	0.6888 (2)	0.0400 (6)	
H5	0.6098	0.9166	0.7125	0.048*	
C6	0.5730 (4)	0.6794 (3)	0.7109 (2)	0.0362 (6)	
H6	0.6825	0.6174	0.7497	0.043*	
C7	0.4555 (3)	0.5897 (3)	0.6759 (2)	0.0303 (5)	
C8	0.6908 (4)	0.3131 (3)	0.8091 (2)	0.0328 (5)	
C9	0.6355 (4)	0.3133 (3)	0.9291 (2)	0.0378 (6)	
H9	0.5055	0.3396	0.9548	0.045*	
C10	0.7722 (4)	0.2748 (4)	1.0114 (2)	0.0405 (6)	
H10	0.7343	0.2740	1.0921	0.049*	
C11	0.9646 (4)	0.2375 (3)	0.9731 (2)	0.0335 (6)	
C12	1.0216 (4)	0.2340 (4)	0.8540 (3)	0.0447 (7)	
H12	1.1518	0.2070	0.8288	0.054*	
C13	0.8857 (4)	0.2704 (4)	0.7719 (3)	0.0461 (7)	
H13	0.9246	0.2663	0.6915	0.055*	
C14	1.1122 (4)	0.2104 (4)	1.0605 (3)	0.0450 (7)	

### Atomic displacement parameters (Å^2^)

**Table:**

	*U*^11^	*U*^22^	*U*^33^	*U*^12^	*U*^13^	*U*^23^
S1	0.0441 (4)	0.0305 (4)	0.0495 (4)	−0.0120 (3)	−0.0242 (3)	−0.0013 (3)
F1	0.0508 (12)	0.0796 (15)	0.0942 (17)	0.0002 (10)	−0.0385 (11)	−0.0105 (12)
F2	0.0770 (15)	0.134 (2)	0.0508 (12)	−0.0459 (15)	−0.0335 (10)	0.0265 (12)
F3	0.057 (5)	0.0356 (19)	0.078 (4)	−0.0181 (19)	−0.034 (4)	−0.002 (2)
F3A	0.15 (3)	0.034 (3)	0.14 (2)	−0.024 (7)	−0.11 (2)	−0.002 (6)
O1	0.0417 (10)	0.0349 (10)	0.0504 (11)	−0.0126 (8)	−0.0228 (8)	−0.0039 (8)
O2	0.0356 (10)	0.0404 (10)	0.0559 (12)	−0.0100 (8)	−0.0219 (8)	−0.0055 (9)
C1	0.0336 (13)	0.0385 (14)	0.0295 (12)	−0.0123 (10)	−0.0104 (10)	0.0000 (10)
C2	0.0343 (13)	0.0335 (12)	0.0293 (12)	−0.0112 (10)	−0.0075 (9)	−0.0039 (9)
C3	0.0381 (13)	0.0339 (13)	0.0410 (14)	−0.0075 (11)	−0.0138 (11)	−0.0026 (10)
C4	0.0456 (15)	0.0300 (13)	0.0460 (15)	−0.0124 (11)	−0.0143 (12)	−0.0016 (11)
C5	0.0415 (15)	0.0410 (15)	0.0452 (15)	−0.0190 (12)	−0.0141 (12)	−0.0055 (11)
C6	0.0331 (13)	0.0373 (14)	0.0412 (14)	−0.0117 (11)	−0.0143 (11)	−0.0019 (11)
C7	0.0308 (12)	0.0316 (12)	0.0302 (12)	−0.0101 (10)	−0.0078 (9)	−0.0023 (9)
C8	0.0339 (12)	0.0267 (12)	0.0393 (13)	−0.0082 (9)	−0.0143 (10)	−0.0009 (10)
C9	0.0317 (13)	0.0391 (14)	0.0441 (15)	−0.0103 (11)	−0.0066 (11)	−0.0067 (11)
C10	0.0466 (15)	0.0464 (16)	0.0333 (13)	−0.0192 (12)	−0.0066 (11)	−0.0041 (11)
C11	0.0387 (13)	0.0219 (11)	0.0424 (14)	−0.0100 (9)	−0.0145 (11)	−0.0005 (9)
C12	0.0320 (13)	0.0542 (17)	0.0466 (16)	−0.0077 (12)	−0.0078 (11)	−0.0086 (13)
C13	0.0395 (15)	0.0595 (18)	0.0365 (14)	−0.0091 (13)	−0.0037 (11)	−0.0071 (13)
C14	0.0550 (18)	0.0317 (13)	0.0538 (17)	−0.0154 (12)	−0.0258 (14)	0.0006 (12)

### Geometric parameters (Å, º)

**Table:**

S1—C7	1.782 (2)	C5—H5	0.9300
S1—C8	1.783 (2)	C5—C6	1.387 (4)
F1—C14	1.328 (4)	C6—H6	0.9300
F2—C14	1.322 (4)	C6—C7	1.396 (3)
F3—C14	1.343 (8)	C8—C9	1.381 (4)
F3A—C14	1.35 (2)	C8—C13	1.390 (4)
O1—C1	1.218 (3)	C9—H9	0.9300
O2—H2	0.8200	C9—C10	1.384 (4)
O2—C1	1.324 (3)	C10—H10	0.9300
C1—C2	1.477 (3)	C10—C11	1.378 (4)
C2—C3	1.400 (4)	C11—C12	1.375 (4)
C2—C7	1.404 (3)	C11—C14	1.496 (4)
C3—H3	0.9300	C12—H12	0.9300
C3—C4	1.383 (4)	C12—C13	1.380 (4)
C4—H4	0.9300	C13—H13	0.9300
C4—C5	1.381 (4)		
			
C7—S1—C8	101.59 (11)	C13—C8—S1	120.6 (2)
C1—O2—H2	109.5	C8—C9—H9	119.7
O1—C1—O2	122.6 (2)	C8—C9—C10	120.5 (2)
O1—C1—C2	123.3 (2)	C10—C9—H9	119.7
O2—C1—C2	114.1 (2)	C9—C10—H10	120.2
C3—C2—C1	118.3 (2)	C11—C10—C9	119.7 (2)
C3—C2—C7	119.4 (2)	C11—C10—H10	120.2
C7—C2—C1	122.3 (2)	C10—C11—C14	120.1 (2)
C2—C3—H3	119.2	C12—C11—C10	120.3 (2)
C4—C3—C2	121.6 (2)	C12—C11—C14	119.5 (3)
C4—C3—H3	119.2	C11—C12—H12	120.0
C3—C4—H4	120.6	C11—C12—C13	120.0 (3)
C5—C4—C3	118.8 (2)	C13—C12—H12	120.0
C5—C4—H4	120.6	C8—C13—H13	119.9
C4—C5—H5	119.7	C12—C13—C8	120.2 (3)
C4—C5—C6	120.7 (2)	C12—C13—H13	119.9
C6—C5—H5	119.7	F1—C14—F3	97.8 (5)
C5—C6—H6	119.5	F1—C14—F3A	122 (3)
C5—C6—C7	121.1 (2)	F1—C14—C11	113.7 (2)
C7—C6—H6	119.5	F2—C14—F1	104.4 (2)
C2—C7—S1	120.83 (18)	F2—C14—F3	115.3 (6)
C6—C7—S1	120.71 (19)	F2—C14—F3A	91 (3)
C6—C7—C2	118.5 (2)	F2—C14—C11	113.2 (2)
C9—C8—S1	120.1 (2)	F3—C14—C11	111.3 (4)
C9—C8—C13	119.2 (2)	F3A—C14—C11	110.3 (8)
			
S1—C8—C9—C10	178.6 (2)	C8—S1—C7—C2	−165.8 (2)
S1—C8—C13—C12	−179.4 (2)	C8—S1—C7—C6	14.5 (2)
O1—C1—C2—C3	165.0 (3)	C8—C9—C10—C11	0.6 (4)
O1—C1—C2—C7	−13.0 (4)	C9—C8—C13—C12	−2.0 (4)
O2—C1—C2—C3	−13.7 (3)	C9—C10—C11—C12	−1.7 (4)
O2—C1—C2—C7	168.3 (2)	C9—C10—C11—C14	174.9 (2)
C1—C2—C3—C4	−177.4 (2)	C10—C11—C12—C13	1.0 (4)
C1—C2—C7—S1	−2.1 (3)	C10—C11—C14—F1	151.3 (3)
C1—C2—C7—C6	177.6 (2)	C10—C11—C14—F2	32.4 (4)
C2—C3—C4—C5	−0.5 (4)	C10—C11—C14—F3	−99.4 (7)
C3—C2—C7—S1	179.85 (19)	C10—C11—C14—F3A	−68 (4)
C3—C2—C7—C6	−0.4 (4)	C11—C12—C13—C8	0.9 (5)
C3—C4—C5—C6	0.0 (4)	C12—C11—C14—F1	−32.1 (4)
C4—C5—C6—C7	0.3 (4)	C12—C11—C14—F2	−151.0 (3)
C5—C6—C7—S1	179.6 (2)	C12—C11—C14—F3	77.2 (7)
C5—C6—C7—C2	−0.1 (4)	C12—C11—C14—F3A	109 (4)
C7—S1—C8—C9	89.1 (2)	C13—C8—C9—C10	1.2 (4)
C7—S1—C8—C13	−93.5 (2)	C14—C11—C12—C13	−175.6 (3)
C7—C2—C3—C4	0.7 (4)		

### Hydrogen-bond geometry (Å, º)

**Table:**

*D*—H···*A*	*D*—H	H···*A*	*D*···*A*	*D*—H···*A*
O2—H2···O1^i^	0.82	1.86	2.677 (3)	175
C6—H6···F3^ii^	0.93	2.59	3.319 (10)	136
C6—H6···F3*A*^ii^	0.93	2.50	3.294 (16)	144

Symmetry codes: (i) −*x*, −*y*+1, −*z*+1; (ii) −*x*+2, −*y*+1, −*z*+2.
